# Cervical Cancer Histology, Staging and Survival before and after Implementation of Organised Cervical Screening Programme in Poland

**DOI:** 10.1371/journal.pone.0155849

**Published:** 2016-05-19

**Authors:** Andrzej Nowakowski, Marek Cybulski, Irmina Buda, Iwona Janosz, Katarzyna Olszak-Wąsik, Piotr Bodzek, Andrzej Śliwczyński, Zbigniew Teter, Anita Olejek, Włodzimierz Baranowski

**Affiliations:** 1 Department of Gynaecology and Oncological Gynaecology, Military Institute of Medicine, Warsaw, Poland; 2 Department of Biochemistry and Molecular Biology, Medical University of Lublin, Lublin, Poland; 3 Department of Obstetrics, Gynaecology and Oncological Gynaecology, Medical University of Silesia, Bytom, Poland; 4 National Health Fund, Central Office, Warsaw, Poland; State University of Maringá/Universidade Estadual de Maringá, BRAZIL

## Abstract

A population-based organised cervical cancer screening programme (OCCSP) was introduced in Poland in 2006. In this study we have aimed to analyse whether selected parameters related to invasive cervical cancer (ICC) of patients diagnosed in two distant gynaecological oncology centres changed after the first screening round of the programme run between 2006–2008. We have run a retrospective cross-sectional analysis of 189 women diagnosed with ICC between 2002–2005 (directly before introduction of the programme) and 165 patients diagnosed between 2009–2012 (just after the first screening round of the programme) and compared their age at diagnosis, histology, stage of tumours and overall survival (OS). Mean age of patients diagnosed in years 2002–2005 and 2009–2012 was 52.1 and 52.6 years respectively. Squamous cell carcinomas constituted 90.5% and 86.1% of tumours diagnosed in years 2002–2005 and 2009–2012 respectively and the rest of tumours had glandular and other histologies. 74.5% and 61.0% of women diagnosed in years 2002–2005 and 2009–2012 respectively had early ICC (FIGO—International Federation of Gynaecology and Obstetrics stages I-IIA) and the rest had advanced disease (FIGO IIB-IV). We have noticed no significant differences in mean age of patients, histology of tumours and OS of patients with ICC diagnosed before and after the first screening round of OCSSP in Poland. Advanced stages of ICC were more commonly diagnosed after the introduction of OCSSP. Changes only in some clinical parameters of patients with ICC were noticed before and after the first screening round of OCSSP in Poland but OS of patients remained the same.

## Introduction

Cytology-based cervical cancer screening has a potential to greatly reduce both incidence of and mortality from invasive cervical cancer (ICC) [1]. Very recent data confirm that effective cervical cancer screening programmes reduce incidence of squamous cell carcinoma (SCC) but not adenocarcinoma (ADC) of the cervix [2]. Also, women participating in screening are diagnosed at earlier stages of invasive disease [3]. As a result, introduction of population-based screening programmes in the Nordic countries enabled extended survival of patients with ICC [4].

According to EUCAN data for 2012 [5] Poland ranks 17th and 11th highest in Europe respectively for ICC incidence and mortality with Age Standardised Incidence and Mortality Rates of 15.3 and 7.4/100 000. This corresponds to 3513 new cases and 1858 deaths related to ICC annually [5]. 1-year, 3-year and 5-year prevalence is estimated at: 2971, 7497, 10846 cases respectively. ICC was the most common cancer in Polish women in 1960-ies but both ICC incidence and mortality has been steadily decreasing since then with no evident changes in dynamics of the trends over the last decade [6]. ICC constituted 3.72% of all cancers diagnosed in Polish women and was responsible for 3.98% of all cancer-related deaths in 2013 [7].

Organised cervical cancer screening programme (OCCSP) in Poland was introduced in 2006, and within years 2006–2008 all eligible women aged 25–59 years received a written invitation to undergo a Pap test within the first screening round of the programme. The programme was implemented in the whole country in 2006 with full availability of free-of-charge smears in every gynaecological clinic possessing a contract with the National Health Fund. Complete data registration in a central electronic database began from 1^st^ January 2007. Through the whole course of the programme the coverage rate of the organised screening fluctuated between 21.3% and 26.8% [6]. We have recently described the course of the programme in details and suggested that it has had no evident impact on short-term incidence and mortality trends of cervical cancer in Poland [6]. This might be related to low coverage of the OCCSP and only partial adherence of the programme to European Guidelines. However despite stable and low coverage of registered invitational Pap smears in the OCCSP, questionnaire-based data suggest that participation in cervical cancer screening (either organised or opportunistic) increased markedly between 2004 and 2011 [8,9]. It may reflect increased awareness related to introduction of the OCCSP, accompanying social campaigns and education of women who undergo Pap testing but outside the organised programme in the so-called opportunistic screening [10].

To further investigate if implementation of organised cervical screening programme in Poland had a general impact on cervical cancer in our country, we have analysed histology, staging and survival of patients diagnosed with ICC before introduction and after one full round of the OCCSP.

## Material and Methods

### Design of the study

We conducted a retrospective cross-sectional study of patients with ICC diagnosed and treated at two distant gynaecological oncology centres in Poland: the Department of Gynaecology and Oncologic Gynaecology, Military Institute of Medicine in Warsaw and the Department of Obstetrics, Gynaecology and Oncologic Gynaecology, Medical University of Silesia in Bytom. These two institutions were selected since they provide specialised gynaecological oncology services for both urban and rural populations of two distant regions of Poland with the highest number of inhabitants: Mazovian and Silesian districts. Therefore cohorts of patients treated for ICC at these centres should be fairly representative for patients diagnosed with ICC in the country.

### Study groups and methods

Three hundred and fifty four women diagnosed with ICC were included in the study and composed two cohorts. The first cohort consisted of 189 patients diagnosed in years 2002–2005 (before programme initiation) and the second one included 165 patients diagnosed in the period of 2009–2012 (after the first screening round of the programme in years 2006–2008). Analyses were performed for two age groups: < 60 and ≥ 60 years since the upper age limit of women eligible for the OCCSP is 59 years. Diagnosis in all women was obtained before any treatment initiation and every woman was included in the analysis only once. Histology of tumours was classified according to WHO classification [11] and all cases were divided into three histological groups: squamous cell carcinoma (SCC), adenocarcinoma (ADC) and other diagnoses. Clinical stage of ICC was classified based on FIGO (International Federation of Gyneacology and Obstetrics) 1994 classification and according to FIGO stage, cases were classified as early (IA-IIA) and advanced (IIB-IV) disease [12]. All women were followed from the time of diagnosis until death or the end of the follow-up period (February 1^st^, 2015). Overall survival (OS) was calculated in months and was defined as the time interval from the date of primary surgery to the date of death (failure) or to the end of follow-up for women who were alive (censored). Ethical approval to carry out the study was granted by the Research Ethics Committee of Military Medical Institute, Warsaw, Poland. Since it was a retrospective study and some patients were deceased, obtaining written consent was not possible. The use of data on dates of death of individuals included in the study was approved by the authorities of the National Health Fund in Poland. Individual patients’ data are not presented since they cannot be made publicly available in order to protect patient privacy. Anonimised data are presented in a collective manner and the full set of detailed anonimised patients’ data is available from authors upon request.

### Statistical analysis

Normality of continuous variables was analysed using Kolmogorov-Smirnov test and Shapiro-Wilk's W test. Differences between variables with normal distribution were analysed with T test. The Pearson's Chi^2^ test was used for statistical analysis of the relationships between categorical or ordinal variables. The effect of clinicopathologic parameters on patients' survival was assessed by Kaplan-Meier method and log-rank test. A multivariate analysis was performed according to the Cox proportional hazard model. The results with a 2-sided P<0.05 were considered statistically significant. Statistical analyses were done using Statistica ver. 9.0 (StatSoft. Inc., Tulsa, OK 74104, USA) and R software ver. 3.1.2 (http://www.r-project.org/).

## Results

**Fig 1:**
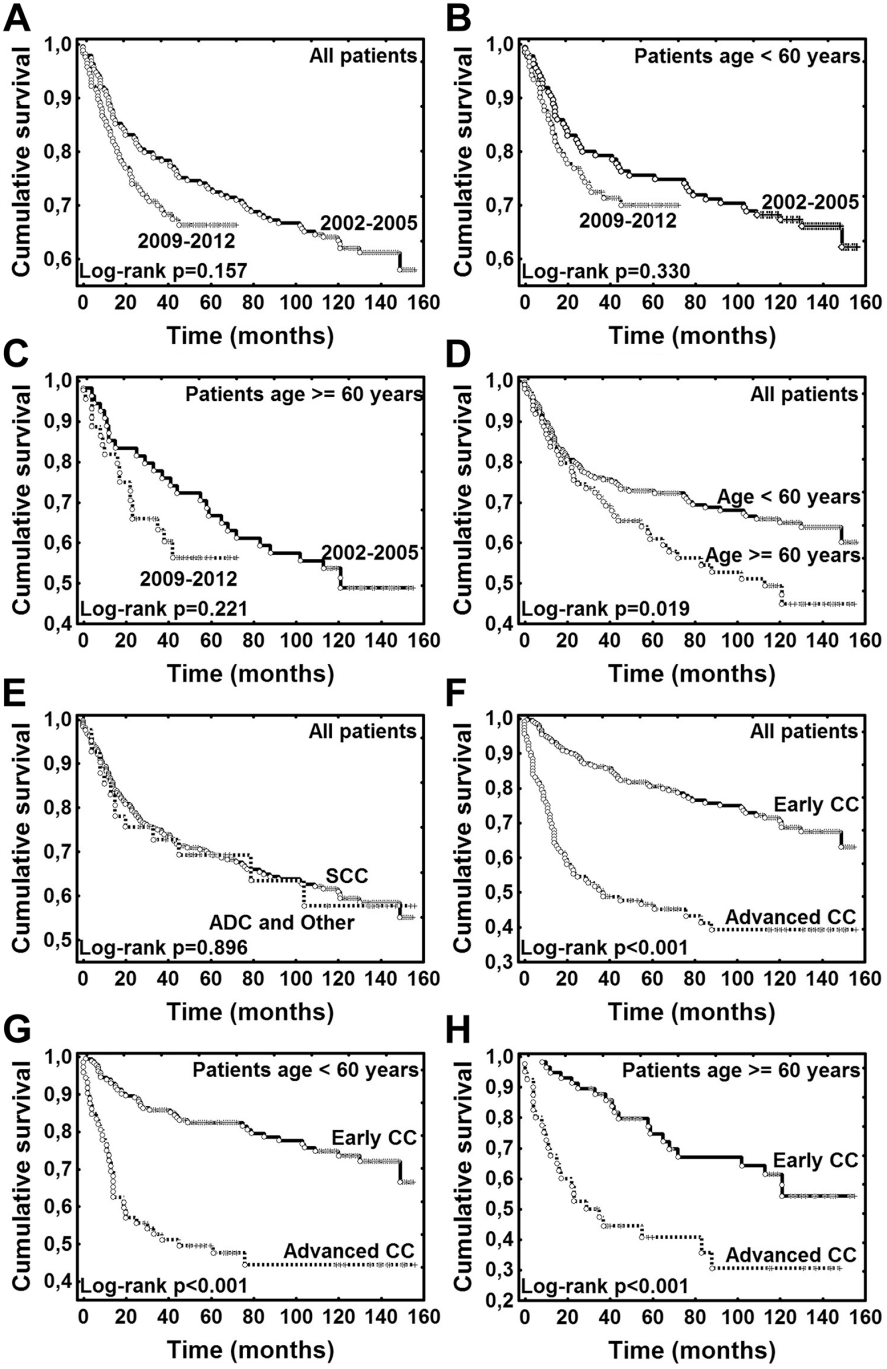
Kaplan-Meier plots for overall survival. (**A)** Women at all ages diagnosed with cervical cancer (CC) before introduction of organised screening (2002–2005) *vs* women diagnosed after one screening round (2009–2012). (**B**) Women < 60 years of age diagnosed with CC before introduction of organised screening (2002–2005) *vs* women diagnosed after one screening round (2009–2012). (**C**) Women ≥ 60 years of age diagnosed with CC before introduction of organised screening (2002–2005) *vs* women diagnosed after one full screening round (2009–2012). (**D**) Women diagnosed with CC before introduction of organised screening and after one screening round according to age at diagnosis: < 60 years *vs* ≥ 60 years. (**E**) Women diagnosed with CC before introduction of organised screening and after one screening round according to histological type of CC: SCC *vs* ADC and other types. (**F**) Women diagnosed with CC before introduction of organised screening and after one screening round according to clinical stage of CC: early *vs* advanced. (**G**) Women < 60 years of age (women diagnosed with CC before introduction of organised screening and after one screening round) according to clinical stage of CC: early *vs* advanced. (**H**) Women ≥ 60 years of age (women diagnosed with CC before introduction of organised screening and after one screening round) according to clinical stage of CC: early *vs* advanced. CC, cervical cancer; SCC, squamous cell carcinoma; ADC, adenocarcinoma; other types include: one case of macrocellular carcinoma, two cases of adenosquamous carcinoma, one case of microcellular and macrocellular carcinoma each, one case of undetermined histological type; Early CC—FIGO (International Society of Gynaecology and Obstetrics 1994 staging system) I-IIa; Advanced CC—FIGO IIb-IV.

**Fig 2:**
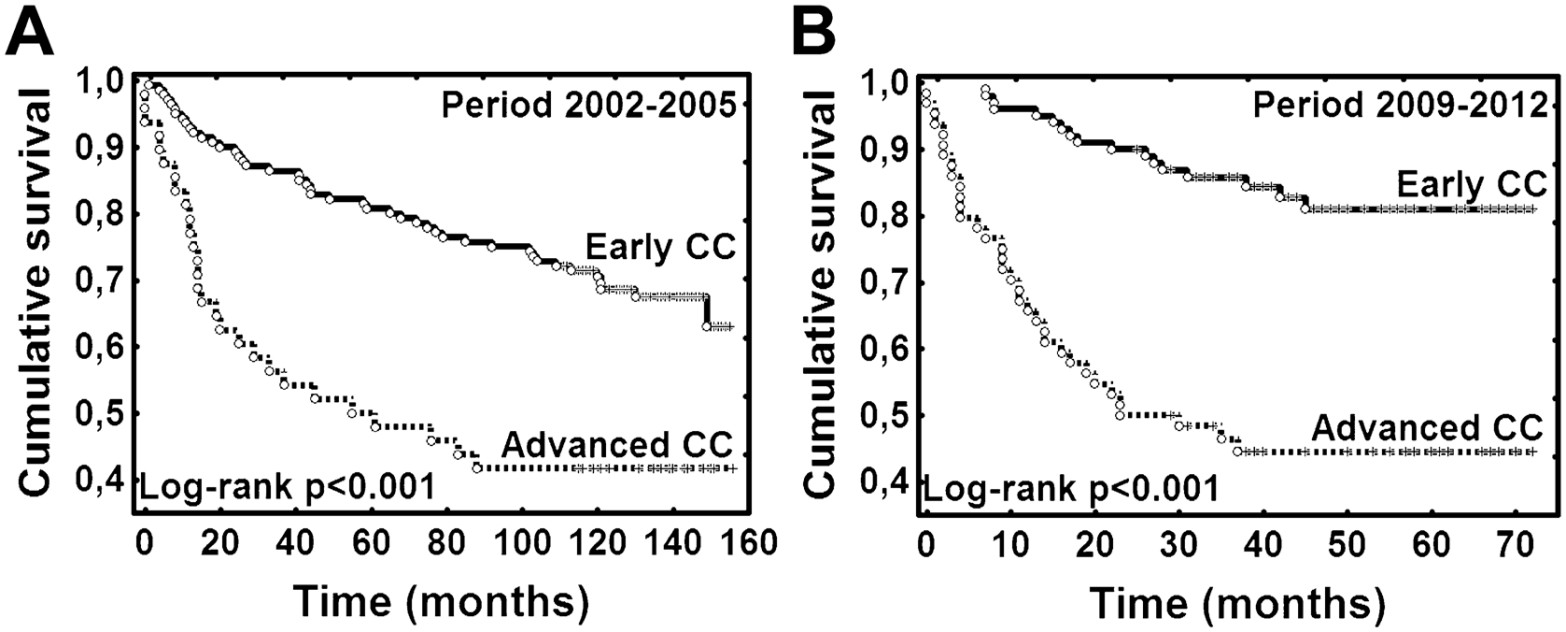
Kaplan-Meier plots for overall survival. Legend: **(A**) Women diagnosed with CC before introduction of organised screening (2002–2005) according to clinical stage of CC: early *vs* advanced. (**B**) Women diagnosed with CC after introduction of organised screening (2009–2015) according to clinical stage of CC: early *vs* advanced. CC, cervical cancer; Early CC—FIGO (International Society of Gynaecology and Obstetrics 1994 staging system) I-IIa; Advanced CC—FIGO IIb-IV.

Patient characteristics are presented in Table 1. There were no statistically significant differences in mean age, age structure (< 60 and ≥ 60 years) and histology between the study groups—before and after one round of organised screening. Advanced ICCs were significantly more frequent in women diagnosed in 2009–2012 compared to period 2002–2005 (39.0% vs 25.5%, p = 0.007, Table 1). Follow-up period ranged from 0 to 156 months (median 57.5 months). At the end of the follow-up (February 1^st^, 2015) 126 (35.6%) women had died. The difference in OS for all patients diagnosed with ICC before introduction of organised screening (2002–2005) *vs* women diagnosed after one full screening round (2009–2012) was not significant; p = 0.157 (log-rank test); (Fig 1A). Also there was no significant differences in OS when comparing the two time periods (2002–2005 *vs* 2009–2012) stratified by age (< 60 and ≥ 60 years, p>0.05); (Fig 1B–1C). In all patients (both periods: 2003–2005 and 2009–2012 combined), OS was significantly higher in younger patients (< 60 years); p = 0.019 (log rank test); (Fig 1D). In all patients (both 2003–2005 and 2009–2012 combined) OS was not different for patients with SCC *vs* ADC and other histologies (p = 0.896, log-rank test); (Fig 1E). OS was significantly lower in women with advanced ICC than those with early ICC in all analysed patients, also when stratified by age (< 60 and ≥ 60 years, p < 0.001, log-rank test); (Fig 1F–1H). Also in patients diagnosed before introduction of the screening (2003–2005) and after one full round (2009–2012) OS was significantly lower in women with advanced ICC than with early (ICC p < 0.001); (Fig 2A–2B). In the multivariate analysis only disease stage was an independent risk factor for OS (hazard ratio (HR) = 3.094, 95% confidence interval (CI) 2.156–4.439, p<0.001); (Table 2). However, non-proportional hazards were detected for age and disease stage (test of proportional hazards, P = 0.031 and P<0.001, respectively). The effect of age was increasing and the effect of stage was declining with time.

**Table 1:** Clinical characteristics of patients with cervical cancer (N = 354).

	Year of diagnosis		P value
	2002-2005(n = 189)	2009-2012(n = 165)	
**Age (years)**			
**Mean ± SD**	52.1 ± 11.8	52.6 ± 12.4	0.677^a^
**Median (range)**	51.0 (26.0–81.0)	52.0 (26.0–89.0)	
	N (%)	N (%)	
**Age group (years)**			
**< 60**	135 (71.4%)	121 (73.3%)	0.689^b^
**≥ 60**	54 (28.6%)	44 (26.7%)	
**Histological subtype**			
**SCC**	171 (90.5%)	142 (86.1%)	0.152^b^
**ADC**	17 (9.00%)	18 (10.9%)	
**Other**	1^c^ (0.5%)	5^d^ (3.0%)	
**SCC**	171 (90.5%)	142 (86.1%)	0.195^b^
**ADC and other**	18 (9.5%)	23 (13.9%)	
	n = 188	n = 160	
**SCC**	171 (91.0%)	142 (88.75%)	0.495^b^
**ADC**	17 (9.0%)	18 (11.25%)	
**Disease stage**			
**Early (FIGO IA-IIA)**	140 (74.5%)	100 (61.0%)	0.007^b^
**Advanced (FIGO IIB-IV)**	48 (25.5%)	64 (39.0%)	
**NA**	1	1	
**Patient survival**			
**Alive**	116 (61.4%)	112 (67.9%)	-
**Deceased**	73 (38.6%)	53 (32.1%)	

^a^ t test;

^b^ Pearson chi^2^ test; SCC, squamous cervical cancer; ADC, cervical adenocarcinoma;

^c^ one case of macrocellular carcinoma;

^d^ two cases of adenosquamous carcinoma, one case of microcellular and macrocellular carcinoma each, one case of undetermined histological type; NA, not available.

**Table 2:** Multivariate analysis (Cox proportional hazard model) of risk factors for overall survival—both analysed groups combined; n = 352 (2 cases deleted due to missing disease stage).

Variable	HR	95% CI for HR	P value
**Age**			
**< 60 years**	1.000	Reference	0.069
**≥ 60 years**	1.408	0.974–2.037	
**Period of diagnosis**			
**2002–2005**	1.000	Reference	0.402
**2009–2012**	1.181	0.801–1.742	
**Histology**			
**SCC**	1.000	Reference	0.564
**ADC and other**^a^	0.848	0.484–1.485	
**Disease stage**			
**Early**	1.000	Reference	<0.001
**Advanced**	3.094	2.156–4.439	

HR, hazard ratio; CI, confidence interval; SCC, squamous cervical cancer; ADC, cervical adenocarcinoma;

^a^ two cases of macrocellular carcinoma, two cases of adenosquamous carcinoma, one case of microcellular, one case of undetermined histological type

## Discussion

ICC incidence and mortality have been decreasing for several decades in Poland however, as we have recently shown, there was no significant changes of the trends related to a recent introduction of the OCCSP in 2006/2007 [6]. The coverage rate of the OCCSP has been low at around ~20–25% and stable but there has been an increase in participation in opportunistic screening [8,9] which might be an indirect effect of increased awareness related to information and educational campaigns led in the OCCSP.

In our study we further investigated whether the first full round (three years) of implementation of the OCCSP had an impact on several parameters related to ICC in Poland such as: age at diagnosis, histology of tumours, stage of the disease at the time of diagnosis and survival of patients. To do this, we compared these parameters in two cohorts of women diagnosed at two distant gynaecological oncology centres in two four-year periods: 1) directly before implementation of the OCCSP (2002–2005) and 2) after one full screening round (2009–2012). We have revealed that there are no significant differences in the mean age of patients diagnosed before and after implementation of the organised screening. The median age of patients in our cohorts (51.0 and 52.0 years respectively for cohorts diagnosed before and after implementation of screening) is very close to that obtained in another cohort of patients diagnosed with ICC in a comparable period (51.0 years) [13] in Poland which confirms credibility of our results. Without existence of screening, age at diagnosis would be mainly influenced by natural risk factors such as exposure to human papillomavirus (HPV), sexual activities, smoking and contraception use. Although some data indicate influence of the screening on mean age of patients with ICC [14], our data indicate no impact of implementation of the OCCSP on this parameter over a short time in Poland.

The two most common histological types of ICC are: SCC and ADC. ADC constitutes around 5% of all ICC worldwide [15] however in some countries, ADC relative rate is increasing and reached 25% [14]. Such trends are typical for countries with very effective cytological screening and have been noted for USA, some provinces of Canada, the Nordic countries and parts of Italy [16]. This is because the screening has been effective primarily at preventing SCC with a minor potential to decrease incidence of invasive ADC [17]. While cytology enables early detection and treatment of SCC precursors, identification of adenocarcinoma *in situ* (AIS), a direct precursor of invasive ADC, by routine cytology and colposcopy is less effective, and is influenced by the variation in the quality and coverage of cytological screening [18–22]. In our study we have noticed a drop in the rates of SCC and an increase in ADC when comparing the cohorts of patients diagnosed with ICC before implementation and after one round of the OCCSP but the difference was not significant. It is possible that the difference would reach significance level over a longer observation period and using the data from larger cohorts.

Histology of ICC did not influence survival in both combined cohorts (before and after one screening round) in our study. There are conflicting literature data on this subject; most indicating worse prognosis and survival, higher relapse and mortality rates in patients with ADC [23–27] and other equal values for SCC and ADC and clear cell carcinoma [14,28]. Some international guidelines indicate that glandular histology may be a risk factor of recurrence [29]. In our centres ADC in patients with ICC is also regarded as a risk factor and the treatment is more aggressive e.g. even radically operated women with ADC undergo adjuvant therapy. This might be responsible for similar OS of patients with SCC and those with ADC combined with and other ICC types in our study.

We have found that women over the screening age (≥ 60 years) diagnosed with ICC in Poland have a worse OS than younger ones. Very recent data from Sweden also confirm a worse prognosis of patients aged 65 and above which is related to the late diagnosis at advanced stages [30]. Also, our data indicating that the stage at diagnosis is the most important factor influencing survival are concordant with numerous previous observations [12].

The most striking result of our study is a significantly higher proportion of patients diagnosed with advanced ICC (FIGO IIB-IV) after implementation of one round of the OCCSP than before. Several factors may be responsible for this finding. Firstly, it is possible that more prevalent cases of advanced but asymptomatic cancers have been detected after implementation of screening. Secondly, although CC staging should be purely clinical [12], upstaging of several patients with distant metastases might have taken place in our institutions due to an increased access to Computer Tomography, Magnetic Resonance and Positrone Magnetic Imaging. Despite more common diagnosis of advanced cases after introduction of OCCSP, the survival was the same, which might be related to advances in treatment modalities of patients with more advanced stages of ICC e.g. novel radiotherapy techniques. Access to modern radiotherapy techniques has increased dramatically in Poland between 2006–2012, mainly due to investments with the National Programme for Fight Against Cancer [31].

We are aware of limitations of our study which include the cross-sectional design and a short time interval between analysed cohorts of patients. However a more comprehensive nationwide analysis based on data collected by the National Cancer Registry (NCR) would be unfeasible at present. The quality of data, especially regarding histology and staging of cancer patients, collected by the NCR is insufficient in Poland to perform such analyses. So our cross-sectional approach was the most feasible at this time to briefly evaluate impact of implementation of the OCCSP on ICC in Poland. OCCSP was introduced less than ten years ago and we wanted to determine its influence on ICC in the country. The selection of two large gynaecological oncology institutions providing services for the two most populated districts in Poland for our analysis should provide a fair approximation of the clinico-pathologic features of women with ICC diagnosed in the whole country. The new edition of the screening programme is being currently prepared and will require enhancements based on the evaluation of the first edition of the OCCSP [6] which should include results of our analysis.

## Conclusions

To conclude, there is no difference in survival of patients diagnosed with ICC before and after implementation of the OCCSP in Poland in 2006/2007. Older women diagnosed with ICC above the screening age have worse OS. Advanced stages of ICC were more commonly diagnosed after introduction of the screening but this finding will require a further insight. There is a non significant shift towards glandular histology being diagnosed more commonly after introduction of OCCSP. Stage of the disease at the time of diagnosis remains the most influential factor for OS in women diagnosed with ICC in Poland.
